# Supplementary material for: Quantitative ultrasound, elastography, and machine learning for assessment of steatosis, inflammation, and fibrosis in chronic liver disease
Source: PLoS One. 2022 Jan 27;17(1):e0262291. doi: 10.1371/journal.pone.0262291 (PMC8794185; doi:10.1371/journal.pone.0262291)
Supplement: S1 Table — Accuracy of each of the eleven features alone and for the best combination of features for classification of steatosis, inflammation, and fibrosis. The best combination depends on the classification task; see Table 2. (DOCX) [file pone.0262291.s001.docx]

**S1 Table. Accuracy of pSWE and QUS features.**

Accuracy of each of the eleven features alone and for the best combination of features for classification of steatosis, inflammation, and fibrosis. The best combination depends on the classification task; see **Table 2**.

|  |  | **AUC-ROC for pSWE and QUS features** | | | | | | | | | | | **AUC-ROC** |
| --- | --- | --- | --- | --- | --- | --- | --- | --- | --- | --- | --- | --- | --- |
| **Pathological features** | **Groups**  **(Size)** | *pSWE* | $\mu_{n}$ Mean | $\mu_{n}$ IQR | 1/$\alpha$ Mean | 1/$\alpha$ IQR | $k$ Mean | $k$ IQR | $1/(\kappa+1)$ Mean | $1/(\kappa+1)$ IQR | Total ACS. | Local ACS. | Best combination |
| Steatosis | 0 vs. ≥ 1  (29/53) | 0.60 | 0.70 | 0.72 | 0.60 | 0.64 | 0.68 | 0.76 | 0.65 | 0.57 | 0.66 | ***0.85*** | **0.90**  (0.89 – 0.91) |
|  | ≤ 1 vs. ≥ 2  (51/31) | 0.63 | 0.67 | 0.71 | 0.58 | 0.60 | 0.62 | 0.63 | 0.63 | 0.59 | 0.57 | ***0.75*** | **0.81**  (0.80 – 0.83) |
|  | ≤ 2 vs. 3  (66/16) | 0.62 | 0.67 | 0.66 | 0.61 | 0.61 | ***0.71*** | 0.67 | 0.64 | 0.61 | 0.62 | 0.69 | **0.78**  (0.77- 0.79) |
| Inflammation | 0 vs. ≥ 1  (8/74) | 0.56 | 0.50 | 0.59 | 0.68 | 0.67 | 0.53 | 0.52 | 0.50 | 0.62 | ***0.75*** | 0.57 | 0.75  (0.73 – 0.76) |
|  | ≤ 1 vs. ≥ 2  (47/35) | 0.62 | 0.58 | 0.59 | 0.60 | 0.62 | ***0.68*** | 0.57 | 0.55 | 0.55 | 0.57 | 0.57 | **0.68**  (0.67 – 0.71) |
|  | ≤ 2 vs. 3  (74/8) | 0.64 | 0.56 | 0.56 | ***0.65*** | 0.58 | 0.64 | 0.53 | 0.58 | 0.58 | 0.57 | 0.63 | 0.69  (0.66 – 0.71) |
| Fibrosis | 0 vs. ≥ 1  (12/70) | 0.66 | 0.50 | 0.55 | 0.53 | 0.65 | ***0.70*** | 0.55 | 0.65 | 0.65 | 0.55 | 0.56 | **0.72**  (0.69 – 0.74) |
|  | ≤ 1 vs. ≥ 2  (25/57) | ***0.77*** | 0.54 | 0.54 | 0.55 | 0.63 | 0.54 | 0.68 | 0.55 | 0.63 | 0.54 | 0.56 | **0.77**  (0.76 – 0.80) |
|  | ≤ 2 vs. ≥ 3  (43/39) | ***0.72*** | 0.57 | 0.60 | 0.56 | 0.58 | 0.59 | 0.58 | 0.57 | 0.68 | 0.61 | 0.63 | **0.77**  (0.76-0.79) |
|  | ≤ 3 vs. 4  (56/26) | ***0.74*** | 0.56 | 0.55 | 0.53 | 0.58 | 0.57 | 0.54 | 0.60 | 0.60 | 0.59 | 0.56 | **0.75**  (0.74-0.77) |

Note: ACS = attenuation coefficient slope. AUC-ROC = area under the receiver operating characteristic curve. Numbers in parentheses are 95% confidence intervals. size = *N/M*, where *N* = number of cases (out of 82 patients) such that pathological feature ≤ *x* (= 0, 1, 2, or 3) and *M* = 82 – *N*; *pSWE =* point shear wave elasticity; $\mu_{n}$ = mean intensity normalized by its maximal value; $1/\alpha$ = reciprocal of the scatterer clustering parameter; $k$ = coherent-to-diffuse signal ratio; $1/(\kappa+1)$ = diffuse-to-total signal power ratio; IQR = inter-quartile range.
