# Supplementary material for: Quantitative ultrasound, elastography, and machine learning for assessment of steatosis, inflammation, and fibrosis in chronic liver disease
Source: PLoS One. 2022 Jan 27;17(1):e0262291. doi: 10.1371/journal.pone.0262291 (PMC8794185; doi:10.1371/journal.pone.0262291)
Supplement: S2 Table — Anova (AOV) test if Shapiro-Wilk (S-W) test succeeded (p-value > 1) or else Kruskal-Wallis (K-S) tests with post-hoc multiple comparison tests with Bonferroni-Holm adjustment of p-values, based on t-test in the former case, or Wilcoxon rank sum test in the latter case. (DOCX) [file pone.0262291.s002.docx]

**S2 Table. ANOVA tests and post-hoc multiple comparisons when applicable for each of these features.**

Anova (AOV) test if Shapiro-Wilk (S-W) test succeeded (*p*-value > 1) or else Kruskal-Wallis (K-S) tests with post-hoc multiple comparison tests with Bonferroni-Holm adjustment of *p*-values, based on t-test in the former case, or Wilcoxon rank sum test in the latter case.

| **Pathological features** | **Groups**  **(Size)** | *pSWE* | $\mu_{n}$ Mean | $\mu_{n}$ IQR | $1/\alpha$ Mean | $1/\alpha$ IQR | $k$ Mean | $k$ IQR | $1/(\kappa+1)$ Mean | $1/(\kappa+1)$ IQR | Total ACS | Local ACS |
| --- | --- | --- | --- | --- | --- | --- | --- | --- | --- | --- | --- | --- |
| **S-W (*p*-value)** |  | 1.5e-6  (K-S) | 0.045  (K-S) | **0.18**  (AOV) | 1.4e-4  (K-S) | 5.7e-8  (K-S) | 6.4e-7  (K-S) | 1.4e-11  (K-S) | 2.6e-7  (K-S) | 2.3e-4  (K-S) | **0.19**  (AOV) | **0.13**  (AOV) |
| Steatosis | 0, 1, 2, 3  (29/22/15/16) | 0.15 | **0.003** | 0.18 | 0.79 | 0.074 | **0.004** | 0.52 | **0.006** | 0.59 | **0.009** | **1.1e-7** |
| Stat. diff. |  |  | **0/1-2-3** |  |  |  | **0/1-2-3** |  | **0/3** |  | **0/3** | **0/1-2-3,1/3** |
| Inflammation | 0, 1, 2, 3  (8/39/27/8) | 0.055 | 0.51 | 0.52 | **0.044** | 0.23 | 0.29 | 0.64 | 0.41 | 0.43 | 0.35 | 0.38 |
| Stat. diff. |  |  |  |  | **none** |  |  |  |  |  |  |  |
| Fibrosis | 0, 1, 2, 3, 4  (12/13/18/13/26) | **8.2e-5** | 0.99 | 0.95 | 0.35 | 0.53 | 0.30 | 0.90 | 0.24 | 0.33 | 0.39 | 0.37 |
| Stat. diff. |  | **0/4,1/2-3-4,2/3** |  |  |  |  |  |  |  |  |  |  |

Note: size indicate the cardinality of each factor group (out of 82 patients); ACS = attenuation coefficient slope. *pSWE =* point shear wave elasticity$\mu_{n}$ = mean intensity normalized by its maximal value; $1/\alpha$ = reciprocal of the scatterer clustering parameter; $k$ = coherent-to-diffuse signal ratio; $1/(\kappa+1)$ = diffuse-to-total signal power ratio; IQR = inter-quartile range; Stat. diff. = statistically different (*p* < 0.05) groups under appropriate post-hoc multiple comparison.
