# Supplementary material for: Quantitative ultrasound, elastography, and machine learning for assessment of steatosis, inflammation, and fibrosis in chronic liver disease
Source: PLoS One. 2022 Jan 27;17(1):e0262291. doi: 10.1371/journal.pone.0262291 (PMC8794185; doi:10.1371/journal.pone.0262291)
Supplement: S3 Table — All features taken as input to random forests and of the best combination of features for classification of steatosis, inflammation, and fibrosis. The best combination of features depends on the classification task; see Table 2. (DOCX) [file pone.0262291.s003.docx]

**S3 Table.** **Accuracy of all eleven features obtained with 0.638+ bootstrap method.**All features taken as input to random forests and of the best combination of features for classification of steatosis, inflammation, and fibrosis. The best combination of features depends on the classification task; see **Table 2**.

|  |  | **AUC-ROC** | |
| --- | --- | --- | --- |
| **Pathological features** | **Groups**  **(Size)** | **All features** | **Best combination** |
| Steatosis | 0 vs. ≥ 1  (29/53) | 0.86 | **0.90**  (0.89 – 0.91) |
|  | ≤ 1 vs. ≥ 2  (51/31) | 0.77 | **0.81**  (0.80 – 0.83) |
|  | ≤ 2 vs. 3  (66/16) | 0.74 | **0.78**  (0.77- 0.79) |
| Inflammation | 0 vs. ≥ 1  (8/74) | 0.63 | **0.75**  (0.73 – 0.76) |
|  | ≤ 1 vs. ≥ 2  (47/35) | 0.63 | **0.68**  (0.67 – 0.71) |
|  | ≤ 2 vs. 3  (74/8) | 0.50 | **0.69**  (0.66 – 0.71) |
| Fibrosis | 0 vs. ≥ 1  (12/70) | 0.62 | **0.72**  (0.69 – 0.74) |
|  | ≤ 1 vs. ≥ 2  (25/57) | 0.70 | **0.77**  (0.76 – 0.80) |
|  | ≤ 2 vs. ≥ 3  (43/39) | 0.72 | **0.77**  (0.76-0.79) |
|  | ≤ 3 vs. 4  (56/26) | 0.68 | **0.75**  (0.74-0.77) |

Note: AUC-ROC = area under the receiver operating characteristic curve. Numbers in parentheses are 95% confidence intervals. size = *N/M*, where *N* = number of cases (out of 82 patients) such that pathological feature ≤ *x* (= 0, 1, 2, or 3) and *M* = 82 – *N*.
