# Supplementary material for: Quantitative ultrasound, elastography, and machine learning for assessment of steatosis, inflammation, and fibrosis in chronic liver disease
Source: PLoS One. 2022 Jan 27;17(1):e0262291. doi: 10.1371/journal.pone.0262291 (PMC8794185; doi:10.1371/journal.pone.0262291)
Supplement: S5 Table — The reported importance is represented by the mean decrease in accuracy, where a higher value corresponds to a higher importance. (DOCX) [file pone.0262291.s005.docx]

**S5 Table. Importance of each parameter within the multi-parameter combinations appearing in Table 2 for the various dichotomous classification tasks.**

The reported importance is represented by the mean decrease in accuracy, where a higher value corresponds to a higher importance.

| **Pathological features** | **Groups** | *pSWE* | $\mu_{n}$ Mean | $\mu_{n}$ IQR | $1/\alpha$ Mean | $1/\alpha$ IQR | $k$ Mean | $k$ IQR | $1/(\kappa+1)$ Mean | $1/(\kappa+1)$ IQR | Total ACS | Local ACS |
| --- | --- | --- | --- | --- | --- | --- | --- | --- | --- | --- | --- | --- |
| Steatosis | S0 vs. S1-3 |  |  |  |  |  |  | 25.1 |  | 5.4 |  | 41.9 |
|  | S0-1 vs. S2-3 | 4.9 |  |  |  |  |  | 3.7 |  |  |  | 26.9 |
|  | S0-2 vs. S3 |  | 2.5 |  |  |  | 3.8 |  |  |  |  | 13.5 |
| Inflammation | A0 vs. A1-3 |  |  |  |  |  |  |  |  |  | -4.7 |  |
|  | A0-1 vs. A2-3 |  |  |  |  |  | 28.5 |  |  |  |  |  |
|  | A0-2 vs. A3 | 8.7 |  |  |  | 6.7 |  |  |  |  |  |  |
| Fibrosis | F0 vs. F1-4 | 2.3 |  |  |  |  | 16.4 |  | 15.3 |  |  |  |
|  | F0-1 vs. F2-4 | 31.9 |  | 5.5 |  |  |  | 10.9 |  | 2.6 |  |  |
|  | F0-2 vs. F3-4 | 29.1 |  | 5.4 |  |  |  |  |  | 10.8 |  |  |
|  | F0-3 vs. F4 | 24.1 |  | -4.0 |  |  |  |  |  | 3.0 | -2.2 |  |

Note: ACS = attenuation coefficient slope. *pSWE =* point shear wave elasticity $\mu_{n}$ = mean intensity normalized by its maximal value; $1/\alpha$ = reciprocal of the scatterer clustering parameter; $k$ = coherent-to-diffuse signal ratio; $1/(\kappa+1)$ = diffuse-to-total signal power ratio; IQR = inter-quartile range.
