# Supplementary material for: Quantitative ultrasound, elastography, and machine learning for assessment of steatosis, inflammation, and fibrosis in chronic liver disease
Source: PLoS One. 2022 Jan 27;17(1):e0262291. doi: 10.1371/journal.pone.0262291 (PMC8794185; doi:10.1371/journal.pone.0262291)
Supplement: S6 Table — The same combinations of parameters as in Table 2 were tested with SVM. The parameter γ of radial basis functions (RBF) was set to the reciprocal of the number of parameters (default value with R package “e1071”); the degree of RBFs was the default value 3; the cost C was varied according to powers of 2 for each combination of parameters and the best resulting AUC-ROC is reported. (DOCX) [file pone.0262291.s006.docx]

**S6 Table:** **Accuracy of shear wave elasticity alone and in combination with quantitative ultrasound (QUS) features for classification of steatosis, inflammation, and fibrosis, based on support vector machine (SVM) models.**

The same combinations of parameters as in Table 2 were tested with SVM. The parameter γ of radial basis functions (RBF) was set to the reciprocal of the number of parameters (default value with R package “e1071”); the degree of RBFs was the default value 3; the cost *C* was varied according to powers of 2 for each combination of parameters and the best resulting AUC-ROC is reported.

| **Pathological features** | **Groups** | **Size** | **AUC-ROC**  **pSWE only** | **AUC-ROC**  **Multi-parameter** | **Parameters** |
| --- | --- | --- | --- | --- | --- |
| Steatosis | S0 vs. S1-3 | 29/53 | 0.58  (0.56 – 0.59) | 0.91  (0.90 – 0.91) | $k$ IQR + 1/($\kappa$ + 1) IQR  + Local ACS |
|  | S0-1 vs. S2-3 | 51/31 | 0.59  (0.57 – 0.61) | 0.81  (0.80 – 0.82) | $k$ IQR + *pSWE*  + Local ACS |
|  | S0-2 vs. S3 | 66/16 | 0.59  (0.57 – 0.61) | 0.73  (0.71- 0.75) | $k$ Mean + $\mu_{n}$ Mean  + Local ACS |
| Inflammation | A0 vs. A1-3 | 8/74 | 0.49  (0.48 – 0.49) | 0.76  (0.72 – 0.76) | Total ACS |
|  | A0-1 vs. A2-3 | 47/35 | 0.62  (0.61 – 0.63) | 0.64  (0.62 – 0.66) | $k$ Mean |
|  | A0-2 vs. A3 | 74/8 | 0.62  (0.58 – 0.63) | 0.64  (0.60 – 0.66) | *pSWE* + $1/\alpha$ IQR |
| Fibrosis | F0 vs. F1-4 | 12/70 | 0.64  (0.62 – 0.65) | 0.66  (0.65 – 0.67) | *pSWE* + $k$ Mean  $+1/(\kappa+1)$Mean |
|  | F0-1 vs. F2-4 | 25/57 | 0.78  (0.77 – 0.79) | 0.78  (0.77 – 0.80) | *pSWE* + $\mu_{n}$ IQR  + $k$ IQR + 1/$( \kappa+1$) IQR |
|  | F0-2 vs. F3-4 | 43/39 | 0.73  (0.72 – 0.75) | 0.75  (0.74-0.77) | *pSWE* + $\mu_{n}$ IQR  $+1/(\kappa+1)$ IQR |
|  | F0-3 vs. F4 | 56/26 | 0.75  (0.73 – 0.76) | 0.75  (0.73-0.77) | *pSWE* + $\mu_{n}$ IQR  $+1/(\kappa+1)$ IQR + Total ACS |

ACS = attenuation coefficient slope. AUC-ROC = area under the receiver operating characteristic curve. Numbers in parentheses are 95% confidence intervals. size = *N/M*, where *N* = number of cases (out of 82 patients) such that pathological feature ≤ *x* (= 0, 1, 2, or 3) and *M* = 82 – *N*; *pSWE =* point shear wave elasticity; $\mu_{n}$ = mean intensity normalized by its maximal value; $1/\alpha$ = reciprocal of the scatterer clustering parameter; $k$ = coherent-to-diffuse signal ratio; $1/(\kappa+1)$ = diffuse-to-total signal power ratio; IQR = inter-quartile range.
