# Supplementary material for: Quantitative ultrasound, elastography, and machine learning for assessment of steatosis, inflammation, and fibrosis in chronic liver disease
Source: PLoS One. 2022 Jan 27;17(1):e0262291. doi: 10.1371/journal.pone.0262291 (PMC8794185; doi:10.1371/journal.pone.0262291)
Supplement: Attachment — Submitted filename: Response to Reviewers.docx [file pone.0262291.s007.docx]

PONE-D-21-21119
Quantitative ultrasound, elastography, and machine learning for assessment of steatosis, inflammation, and fibrosis in chronic liver disease
PLOS ONE

Dear Dr. Tang,

Thank you for submitting your manuscript to PLOS ONE. After careful consideration, we feel that it has merit but does not fully meet PLOS ONE’s publication criteria as it currently stands. Therefore, we invite you to submit a revised version of the manuscript that addresses the points raised during the review process.

Please submit your revised manuscript by Sep 27 2021 11:59PM. If you will need more time than this to complete your revisions, please reply to this message or contact the journal office at [plosone@plos.org](mailto:plosone@plos.org). When you're ready to submit your revision, log on to <https://www.editorialmanager.com/pone/> and select the 'Submissions Needing Revision' folder to locate your manuscript file.

Please include the following items when submitting your revised manuscript:

- A rebuttal letter that responds to each point raised by the academic editor and reviewer(s). You should upload this letter as a separate file labeled 'Response to Reviewers'.
- A marked-up copy of your manuscript that highlights changes made to the original version. You should upload this as a separate file labeled 'Revised Manuscript with Track Changes'.
- An unmarked version of your revised paper without tracked changes. You should upload this as a separate file labeled 'Manuscript'.

If you would like to make changes to your financial disclosure, please include your updated statement in your cover letter. Guidelines for resubmitting your figure files are available below the reviewer comments at the end of this letter.

If applicable, we recommend that you deposit your laboratory protocols in [protocols.io](http://protocols.io) to enhance the reproducibility of your results. Protocols.io assigns your protocol its own identifier (DOI) so that it can be cited independently in the future. For instructions see: <http://journals.plos.org/plosone/s/submission-guidelines#loc-laboratory-protocols>. Additionally, PLOS ONE offers an option for publishing peer-reviewed Lab Protocol articles, which describe protocols hosted on [protocols.io](http://protocols.io). Read more information on sharing protocols at <https://plos.org/protocols?utm_medium=editorial-email&utm_source=authorletters&utm_campaign=protocols>.

We look forward to receiving your revised manuscript.

Kind regards,

Omar Sultan Al-Kadi, PhD
Academic Editor
PLOS ONE

Journal Requirements:

When submitting your revision, we need you to address these additional requirements.

1. Please ensure that your manuscript meets PLOS ONE's style requirements, including those for file naming. The PLOS ONE style templates can be found at

<https://journals.plos.org/plosone/s/file?id=wjVg/PLOSOne_formatting_sample_main_body.pdf> and

<https://journals.plos.org/plosone/s/file?id=ba62/PLOSOne_formatting_sample_title_authors_affiliations.pdf>

**Response: We have reviewed the PLOS ONE's style requirements and followed them carefully. While reviewing the manuscript, we noticed that k and kappa (which have different meanings in the field of QUS) were used incorrectly at some locations in the manuscript and Supplemental Materials. We have made the corrections in version tracking mode. We apologize for this unintentional slip.**

2. Thank you for stating the following in the Acknowledgments Section of your manuscript:

[We thank Mrs. Assia Belblidia, Mrs. Catherine Huet, and Mr. Walid El Abyad for their assistance in patient enrollment. ACUSON S2000 and S3000 ultrasound systems were lent by Siemens Healthineers.

This work was supported by grants from the Canadian Institutes of Health Research (CIHR)-Institute of Nutrition, Metabolism and Diabetes (INMD) (CIHR-INMD #273738 and #301520) and Fonds de recherche du Québec en Santé (FRQS) and Fondation de l'Association des radiologistes du Québec (FARQ) Clinical Research Scholarship – Junior 1 and 2 Salary Award (FRQS-FARQ #26993 and #34939) to An Tang. Junior 1 Salary Award from FRQS (#27127) and research salary from McGill University to Giada Sebastiani.]

We note that you have provided funding information that is not currently declared in your Funding Statement. However, funding information should not appear in the Acknowledgments section or other areas of your manuscript. We will only publish funding information present in the Funding Statement section of the online submission form.

Please remove any funding-related text from the manuscript and let us know how you would like to update your Funding Statement. Currently, your Funding Statement reads as follows:

 [This work was supported by grants from the Canadian Institutes of Health Research (CIHR)-Institute of Nutrition, Metabolism and Diabetes (INMD) (CIHR-INMD #273738 and #301520, <https://cihr-irsc.gc.ca/>) to AT.

This work was also supported by Junior 1 and Junior 2 Clinical Research Scholarships from the Fonds de recherche du Québec en Santé (FRQS, <https://frq.gouv.qc.ca/en/>) and Fondation de l'Association des radiologistes du Québec (FARQ) (FRQS-FARQ #26993 and #34939 to AT; and by a Junior 1 Clinical Research Scholarships from FRQS (#27127) and research salary from McGill University to GS.]

Please include your amended statements within your cover letter; we will change the online submission form on your behalf.

**Response: As suggested, we have removed funding information from the Acknowledgents. The current Funding Statement is complete.**

3. Thank you for stating the following in your Competing Interests section:

[No].

Please complete your Competing Interests on the online submission form to state any Competing Interests. If you have no competing interests, please state "The authors have declared that no competing interests exist.", as detailed online in our guide for authors at <http://journals.plos.org/plosone/s/submit-now>

 This information should be included in your cover letter; we will change the online submission form on your behalf.

**Response: We do not have competing interests. We have included a statement in the revised Cover Letter.**

4. Please include captions for your Supporting Information files at the end of your manuscript, and update any in-text citations to match accordingly. Please see our Supporting Information guidelines for more information: <http://journals.plos.org/plosone/s/supporting-information>.

**Response: We have modified the captions for Supplemental Tables ("S1 Table", "S2 Table", and "S3 Table") instead of "Table S1", "Table S2", and "Table S3", respectively, to comply with the Supporting Information guidelines.**


Reviewers' comments:

Reviewer's Responses to Questions

**Comments to the Author**

1. Is the manuscript technically sound, and do the data support the conclusions?

The manuscript must describe a technically sound piece of scientific research with data that supports the conclusions. Experiments must have been conducted rigorously, with appropriate controls, replication, and sample sizes. The conclusions must be drawn appropriately based on the data presented.

Reviewer #1: Yes

Reviewer #2: Partly

Reviewer #3: Yes

**Response: We thank the reviewers for this favorable assessment of our manuscript. We address concerns raised by Reviewer #2 below.**

2. Has the statistical analysis been performed appropriately and rigorously?

Reviewer #1: Yes

Reviewer #2: Yes

Reviewer #3: Yes

**Response: We thank the reviewers for this favorable assessment of our statistical analysis.**

3. Have the authors made all data underlying the findings in their manuscript fully available?

The [PLOS Data policy](http://www.plosone.org/static/policies.action#sharing) requires authors to make all data underlying the findings described in their manuscript fully available without restriction, with rare exception (please refer to the Data Availability Statement in the manuscript PDF file). The data should be provided as part of the manuscript or its supporting information, or deposited to a public repository. For example, in addition to summary statistics, the data points behind means, medians and variance measures should be available. If there are restrictions on publicly sharing data—e.g. participant privacy or use of data from a third party—those must be specified.

Reviewer #1: No

Reviewer #2: No

Reviewer #3: No

**Response: We now include an Excel file entitled "** **S4 Dataset.xlsx" that provides for each of the 82 patients (in anonymous form) the steatosis and inflammation grades, the fibrosis stage, and the values of the 11 features.**

4. Is the manuscript presented in an intelligible fashion and written in standard English?

PLOS ONE does not copyedit accepted manuscripts, so the language in submitted articles must be clear, correct, and unambiguous. Any typographical or grammatical errors should be corrected at revision, so please note any specific errors here.

Reviewer #1: Yes

Reviewer #2: Yes

Reviewer #3: Yes

**Response: We thank the reviewers for this favorable assessment of our writing.**

5. Review Comments to the Author

Please use the space provided to explain your answers to the questions above. You may also include additional comments for the author, including concerns about dual publication, research ethics, or publication ethics. (Please upload your review as an attachment if it exceeds 20,000 characters)

Reviewer #1: A very nicely structured paper. I have some suggestions that may help the authors to improve the paper.
--Lines 186-192. It's not clear how these parameters were generated. I would suggest clarifying this section

**Response: We thank the reviewer for the comment. In fact, details were provided on lines 195-225. Thus, the brief summary on lines 186-192 might be redundant. We therefore removed the text on lines 186-192 and modified the title of the section spanning lines 193-228 as “Preliminary post-processing”.**

-- NASH-CRN system uses 1a, 1b and 1c. Did you pool these values as 1 and merged with the rest of the group? (METAVIR for other etiologies).

**Response: Yes. To clarify this approach, we added the following sentence in the Histopathology Analysis section:**

**«** **Fibrosis stages 1A, 1B, and 1C in the NASH CRN system were pooled into stage 1.»**

--Using two different ultrasound models (S2000 and S3000) could cause variability. I would suggest adding this factor to the limitations.

**Response: As suggested by the reviewer, we added the following sentence in the Limitations section:**

**«** **Ultrasound images were acquired with two clinical scanner models which could have introduced variability. »**

--I would suggest annotating Fig3 and showing the areas with fibrosis, inflammation and steatosis.

**Response: Please note that the histopathology slides for Figure 3 (renumbered Figure 4) were selected to mirror the cases illustrated in Figure 1. We intentionally focused on steatosis progression in these two figure collages. As suggested by the reviewer, we have added annotations to Figure 3 and the following sentence to the Figure 3 legend:**

**«** **Representative vacuoles of macrovesicular steatosis are indicated by arrows. »**

--I would suggest providing some images from the pSWE exams.

**Response: As suggested, we have added examples of pSWE measurements accompanied by the following figure legend.**

**«Fig 2. Examples of point shear wave elastography (pSWE) measurements.**

**pSWE measurements in 4 different patients : (A) steatosis grade 0, (B) steatosis grade 1, (C) steatosis grade 2, and (D) steatosis grade 3. Green rectangles indicate regions of interest where measurements are performed.»**

--I would suggest using NAS scores and performing analysis if available.
Thank you for considering my recommendations.

**Response: For this pilot study, we developed a model for classification of steatosis grade, inflammation grade, and fibrosis stage in a cohort of patients undergoing liver biopsy for a variety of chronic liver disease. Different scoring systems are used and different histopathological features are recorded depending on the underlying pathology. Of the 82 included subjects, only 6 had NAFLD and 38 had NASH. Hence, we do have the ballooning and lobular inflammation scoring as per NASH CRN system to compute the NAFLD Activity Score (NAS) for these patients, but not for other patients, such as those scored according to the METAVIR system for patients with autoimmune or chronic hepatitis.**

**Thus, while we agree with the reviewer that a model predicting NAS would be highly relevant and desirable, our current cohort and corresponding dataset prevent us from developing such a model.**

Reviewer #2: General Comments:

This study presents a machine learning method for assessing chronic liver disease (CLD) diagnostic parameters (fibrosis, steatosis, inflammation) through ultrasound (US) B-Mode and Elastographic information provided by ultrasound imaging. The study has some merits as the combination of information of fibrosis, steatosis and inflammation for CLD assessment has not yet been evaluated. Also, the statistical analysis of the Results provided is good and properly written. Its novelty is limited to this combination though, as the algorithms used are already known and used widely in the literature. Its flow is sometimes confusing as many aspects referred for the first time are explained in other, later parts of the manuscript. Furthermore, the manuscript has some major flaws that should be considered before publication.

Non-Specific Comments:

1. The study’s text flow is sometimes confusing as many aspects referred for the first time are explained in other, later parts of the manuscript. For example, the histological information section containing Metavir Classification systems for fibrosis and inflammation (activity) should be earlier in the manuscript as there are points that stage-grade information is shown that are unknown to the non-expert reader, and are not explained. For a large part of the manuscript it is not clear whether the Metavir or Ishak classification system is used. Also, Table 2 should be transferred in the Results section as it is early in the Methods section.

**Response: While we understand that a description of the histopathological scoring systems may be helpful earlier in the manuscript, we prefer to respect the traditional order where details of the index test (ultrasound technique) are described first followed by a description of the reference standard (histopathology analysis). Hence, we prefer maintaining the current location as it follows the writing convention.**

**As suggested, we moved Table 2 to the Results section.**

2. The stage-grade notation is a little confusing as usually, in the literature, there is a capital letter ahead of stage-grade number e.g. F≥F2 for fibrosis, S≥S1 for steatosis and A≥A3 for inflammation. Please provide clearer stage-grade class representation.

**Response: We agree that this nomencluture is more self explanatory when discussing multiple histopathological features. As suggested, we modified the nomenclature throughout the manuscript while preserving the same dichotomization schemes:**

**Classification of steatosis grades: S0 vs. S1-3, S0-1 vs. S2-3, S0-2 vs. S3.**

**Classification of inflammation grades: A0 vs. A1-3, A0-1 vs. A2-3, A0-2 vs. A3.**

**Classification of liver fibrosis stages: F0 vs. F1-4, F0-1 vs. F2-4, F0-2 vs. F3-4, F0-3 vs. F4.**

3. The Introduction section needs many additions regarding existing literature. Only clinical information of the CLD is mentioned and minimal information is given on the non-invasive approaches (clinical or A.I. based) for CLD assessment. Many elastographic variants exist (MR- and US-based) that present variable performances in clinical and A.I. related studies that are completely absent on this study. Also, no information is given on other A.I. related works on CLD assessment. What is their performance? Their performance should be discussed in comparison with the literature.

a. Indicative works (much more exist) that the Authors should include and discuss are:

i. Gatos, I., Tsantis, S., et al, (2016), A new computer aided diagnosis system for evaluation of chronic liver disease with ultrasound shear wave elastography imaging. Med. Phys., 43: 1428–1436. doi:10.1118/1.4942383
ii. Gatos, I., Tsantis, S., et al, (2017), A Machine-Learning Algorithm Toward Color Analysis for Chronic Liver Disease Classification, Employing Ultrasound Shear Wave Elastography, Ultrasound in Medicine and Biology, Volume 43, Issue 9, 1797 – 1810. doi:10.1016/j.ultrasmedbio.2017.05.002
iii. Gatos, I., Tsantis, S., et al, (2019), Temporal stability assessment in shear wave elasticity images validated by deep learning neural network for chronic liver disease fibrosis stage assessment. Med. Phys., 46(5): [2298-2309](tel:2298-2309). doi:10.1002/mp.13521
iv. Gatos, I., Drazinos, P., et al, (2020), Comparison of Sound Touch Elastography, Shear Wave Elastography and Vibration-Controlled Transient Elastography in Chronic Liver Disease Assessment using Liver Biopsy as the "Reference Standard". Ultrasound in Medicine and Biology, 46(4):959-971. doi:10.1016/j.ultrasmedbio.2019.12.016.
v. Kagadis, G.C., Drazinos, P., et al, (2020), Deep learning networks on chronic liver disease assessment with fine-tuning of shear wave elastography image sequences. Phys. Med. Biol. [65 215027](tel:65%20215027)
vi. Stoean R, Stoean C, Lupsor M, Stefanescu H and Badea R 2011 Evolutionary-driven support vector machines for determining the degree of liver fibrosis in chronic hepatitis C Artif. Intell. Med. 51 53–65
vii. Wang K et al 2019 Deep learning radiomics of shear wave elastography significantly improved diagnostic performance for assessing liver fibrosis in chronic hepatitis B: a prospective multicentre study Gut [68 729–41](tel:68%20729–41)
viii. Meng D, Zhang L, Cao G, Cao W, Zhang G and Hu B 2017 Liver fibrosis classification based on transfer learning and FCNet for ultrasound images IEEE Access 5 5804–10
ix. Durot I, Akhbardeh A, Sagreiya H, Loening A M and Rubin D L 2020 A new multimodel machine learning framework to improve hepatic fibrosis grading using ultrasound elastography systems from different vendors Ultrasound Med. Biol. 46 26–33

**Response: We agree that several more studies could be cited on US- and MR-based grading of liver fat or staging of liver fibrosis. For the purpose of this original manuscript, we cited some representative work focusing on QUS.**

**As suggested, we have added the following sentence to the Introduction:**

**«More recently, several studies have proposed traditional machine learning [13-16] or deep learning [17-19] techniques combined with shear wave elastography to improve the staging of liver fibrosis.»**

**We have acknowledged the prior work in the Discussion:**

**«Recent works by Durot et al. and Kagadis et al., showed that fibrosis classification accuracy could be further improved, with AUCs in the range of 0.96 to 0.99, by using machine learning techniques such as support vector machines [16] and deep learning techniques applied on shear wave elastography data [19].»**

**References cited:**

**13.	Stoean R, Stoean C, Lupsor M, Stefanescu H, Badea R. Evolutionary-driven support vector machines for determining the degree of liver fibrosis in chronic hepatitis C. Artif Intell Med. 2011;51(1):53-65. Epub 2010/08/03. doi: 10.1016/j.artmed.2010.06.002. PubMed PMID: 20675106.**

**14.	Gatos I, Tsantis S, Spiliopoulos S, Karnabatidis D, Theotokas I, Zoumpoulis P, et al. A new computer aided diagnosis system for evaluation of chronic liver disease with ultrasound shear wave elastography imaging. Med Phys. 2016;43(3):1428-36. Epub 2016/03/05. doi: 10.1118/1.4942383. PubMed PMID: 26936727.**

**15.	Gatos I, Tsantis S, Spiliopoulos S, Karnabatidis D, Theotokas I, Zoumpoulis P, et al. A Machine-Learning Algorithm Toward Color Analysis for Chronic Liver Disease Classification, Employing Ultrasound Shear Wave Elastography. Ultrasound Med Biol. 2017;43(9):1797-810. Epub 2017/06/22. doi: 10.1016/j.ultrasmedbio.2017.05.002. PubMed PMID: 28634041.**

**16.	Durot I, Akhbardeh A, Sagreiya H, Loening AM, Rubin DL. A New Multimodel Machine Learning Framework to Improve Hepatic Fibrosis Grading Using Ultrasound Elastography Systems from Different Vendors. Ultrasound Med Biol. 2020;46(1):26-33. Epub 2019/10/16. doi: 10.1016/j.ultrasmedbio.2019.09.004. PubMed PMID: 31611074; PubMed Central PMCID: PMCPMC6879839.**

**17.	Wang K, Lu X, Zhou H, Gao Y, Zheng J, Tong M, et al. Deep learning Radiomics of shear wave elastography significantly improved diagnostic performance for assessing liver fibrosis in chronic hepatitis B: a prospective multicentre study. Gut. 2019;68(4):729-41. Epub 2018/05/08. doi: 10.1136/gutjnl-2018-316204. PubMed PMID: 29730602; PubMed Central PMCID: PMCPMC6580779.**

**18.	Gatos I, Tsantis S, Spiliopoulos S, Karnabatidis D, Theotokas I, Zoumpoulis P, et al. Temporal stability assessment in shear wave elasticity images validated by deep learning neural network for chronic liver disease fibrosis stage assessment. Med Phys. 2019;46(5):2298-309. Epub 2019/04/01. doi: 10.1002/mp.13521. PubMed PMID: 30929260.**

**19.	Kagadis GC, Drazinos P, Gatos I, Tsantis S, Papadimitroulas P, Spiliopoulos S, et al. Deep learning networks on chronic liver disease assessment with fine-tuning of shear wave elastography image sequences. Phys Med Biol. 2020;65(21):215027. Epub 2020/10/02. doi: 10.1088/1361-6560/abae06. PubMed PMID: 32998480.**

4. The Methods section has also issues as:

a. The Authors compare plain pSWE measurement with all the information processed in the proposed machine learning scheme that is somewhat irrelevant or, at least, should be just complementary. The authors should compare the proposed algorithm’s estimation with the combination of information given by the pSWE measurement and the US B-Mode image by an expert clinician or by other analysis.

**Response: Conceptually, we agree with this comment. Alas, in practice, radiologists do not have imaging features to grade inflammation, diagnose NASH, or reliably stage fibrosis (except when F4 fibrosis or cirrhosis is clearly established). Hence, it would be misleading to compare the combination of QUS and elastography with human interpretation. Instead, we chose to compare the combination of QUS parameters and elastography with the widely accepted technique for assessment of liver fibrosis: elastography.**

b. Only binary class differentiation is presented. Although this is not a major limitation as other class combinations (e.g. ternary or full) may be evaluated due to small sample in certain classes, some could be realized as they may contain significant clinical information (e.g. Mild Fibrosis vs. Significant Fibrosis vs. Cirrhosis, or in other words, F0-F1 vs. F2-F3 vs. F4).

**Response: By reporting binary dichotomizations, we adopted a classification scheme that is the norm in this field of research and clinical practice.**

c. No splitting of data (e.g. 70% training to 30% validation) is provided resulting in no estimation of the robustness of the results.

**Response: We thank the reviewer for the comment. Let us recall that in case of sufficiently large sample size, the performance of a trained classification algorithm is evaluated on an independent test set so as to estimate the true generalization error, assuming that both training and test sets are representative of the target population for a given disease. However, due to the small sample size, we have followed the recommendation of reference [36] by estimating the generalization error in the theoretical framework presented in section 7.11 of [36], which applies to any classifier, including random forests.**

**Based on this reference, we trained and tested random forests for each classification task in the context of the so-called 0.638+ bootstrap method. The goal of this bootstrap method is to provide a fair estimate of the generalization error, in case where a dataset has small sample size thus preventing its partition into disjoint training, validation and test subsets. Each bootstrap results from a sampling with replacement of the entire dataset. The remaining samples are used for testing the random forest that was trained on the bootstrap. An average of classification error, or false positive or negative rates over several bootstraps (and hence on different training sets - bootstraps) is known to overestimate the generalization error [36]. On the other hand, the so-called training error (*i.e*., obtained when using the entire dataset for both training and testing a random forest) is known to underestimate the generalization error (especially in the case of over-fitting). But an appropriate combination of the (leave-one-out) bootstrap error with the training error yields a fair estimate of the generalization error according to [36]. This is what we did in the current study as reported in the manuscript.**

**To address the reviewer’s comment, we added the following sentence (lines 366-370):**

**“This method, which is recommended when sample size is too small for allowing training-validation-test set split, combines the leave-one-out bootstrap error and the training error with adaptive weights that take into account the amount of over-fitting, thus avoiding over-estimation of the generalization error by the former and under-estimation by the latter.”**

5. The Discussion section is incomplete in terms of comparison with relevant clinical and technical works in the literature that is also absent in the Introduction. That being said, the proposed method shows relatively low performance scores (~0.70-0.80) for fibrosis, steatosis and inflammation. Compared to the literature for fibrosis, these are much lower, especially for the proposed scheme to other machine learning schemes. If the proposed scheme’s performance on CLD assessment is inferior to other A.I. variants processing US imaging information, why should it be published? Regarding Steatosis comparison with other popular parameters in the literature as the Hepatorenal Index (HRI) or the Controlled Attenuation Parameter (CAP) is absent. Regarding inflammation, other studies have discussed that elastography in general, provides no significant clinical information. Is this study’s result a confirmation on this aspect? Please discuss and compare with literature. Also, as there are studies in the literature having deep learning implementations on visualized elastography (e.g. Supersonic’s SWE) with far better results, why should a machine learning scheme such as this be preferred? Please also discuss.

**Response: We agree that there are many other publications on alternative methods for steatosis quantification (such as hepatorenal index and attenuation techniques) or fibrosis quantification (especially elastography techniques). However, the focus of this original manuscript was on a comprehensive approach combining QUS and elastography using random forests to assess all three hallmark features of NASH at once rather than provide a review of the state-of-the-art in this field for each individual hallmark feature, as this would be beyond the scope of the current manuscript.**

**Nevertheless, we agree that acknowledgment of steatosis quantification techniques would strengthen this manuscript. We added the following sentence in the Discussion to acknowledge the state of the art in liver fat quantification using ultrasound-based techniques:**

**“Our proposed method compares favorably with recent results reported by Moret et al. for two state-of-the-art techniques: hepatorenal index ratio with AUCs of 0.90 for S0 vs. S1-3, 0.78 for S0-1 vs. S2-3, and 0.73 for S0-2 vs. S3 and controlled attenuation parameter (CAP) with AUCs of 0.93 for S0 vs. S1-3, 0.76 for S0-1 vs. S2-3, and 0.70 for S0-2 vs. S3 [52].”**

**Reference:**

**Moret A, Boursier J, Houssel Debry P, et al. Evaluation of the Hepatorenal B-Mode Ratio and the "Controlled Attenuation Parameter" for the Detection and Grading of Steatosis. Ultraschall Med. 2020.**

Specific Comments:

1. Page 9, Abstract: “This ancillary study to a prospective institutional review-board approved study included 82 patients with non-alcoholic fatty liver disease, chronic hepatitis B or C virus, or autoimmune hepatitis.”

Bad English here. Also, there are some aspects that are unclear. For example, the use of the word «prospective» when in the Methods section you mention that this is a retrospective study. Also, i don’t understand the «ancillary» part that this study fulfills and is mentioned also in the Methods section. Please explain.

**Response: We previously performed a diagnostic imaging trial comparing ultrasound-based and magnetic resonance-based elastography techniques using liver biopsy as the reference standard. The study protocol was announced on a Clinical Trial registry (https://clinicaltrials.gov/ct2/show/NCT02044523) and the results have already been published.

Reference:**

**Lefebvre T, Wartelle-Bladou C, Wong P, Sebastiani G, Giard JM, Castel H, et al. Prospective comparison of transient, point shear wave, and magnetic resonance elastography for staging liver fibrosis. Eur Radiol. 2019;29(12):6477-88. Epub 2019/07/07. doi: 10.1007/s00330-019-06331-4. PubMed PMID: 31278577.**

**From the data acquired in that prospective trials, we performed a retrospective study on quantitative ultrasound data acquired. Since the objectives of distinct from those of the main trial, this is an ancillary study.**

**We politely believe that the wording used in the first two paragraphs of the Materials and Methods section is appropriate, accurately depicts the work performed, and is properly referenced:**

***«*** ***Study design and subjects***

***This is a retrospective, cross-sectional ancillary study to an imaging trial (ClinicalTrials.gov Identifier No. NCT02044523). This study was approved by the institutional review board of the two participating institutions, Centre hospitalier de l’Université de Montréal and McGill University Health Centre. All patients gave written informed consent.***

***The prior cross-sectional imaging trial compared diagnostic accuracy of ultrasound-based and magnetic resonance-based elastography techniques using liver biopsy as the reference standard [6]. For this ancillary study, QUS and elastography measurements were used to develop models for classification of steatosis grade, inflammation grade, and fibrosis stage.»***

Reviewer #3: This paper presented a machine learning method that used QUS and pSWE to classify steatosis grade, inflammation grade, and fibrosis stage in the CLD patient population. Histopathology was used as the gold standard. Results indicated that the addition of QUS was superior than using SWE alone.
The paper was clearly written and easy to follow. The topic is of interest to the medical ultrasound community.
A few comments below:
1. One major weakness of the paper is that only Random Forrest (RF) was used. There is no other machine learning method to compare the RF results against to. It’s well known in the SWE community that due to the current challenge of high variability in SWE acquisition and measurement, SWE alone is not a reliable measurement for inflammation, fibrosis and steatosis grading. Due to the reason above, it is almost expected that combining QUS and SWE will result in improved assessment, therefore without additional method(s) to compare against it is difficult to understand the efficacy of the RF method presented here.
Recommend adding at least one additional machine learning method.

**Response: We thank the reviewer for the suggestion. However, the goal of the submitted study was not to design an optimal machine learning model for steatosis, inflammation and fibrosis dichotomous classification in the clinical context of NASH, but rather to demonstrate the potential value of combining the proposed QUS features with pSWE feature.**

**As for having chosen random forests as classification model, we had written in the manuscript “*Classification tasks were performed with random forests [31]. This statistical learning approach is suitable in the case of relatively small data sets, as random forests avoid over-fitting (i.e., that would be based on too many trees - see Theorem 1.2 and the remark thereafter in [31]), and have very few hyper-parameters to be tuned; i.e., input features and maximum number of terminal nodes (MNTNs) in each tree.*”**

**Let us now recall in more details some advantages of random forests in the presence of datasets with relatively small sample size.**

**1) *Random forests have few hyper-parameters to set, and henceforth are easier to train*.**

**In the implementation that we used (Breiman and Cutler [41]), Cutler *et al* [34] mentions (section 2.4) that: “Although Random Forests have the reputation of working quite well right out of the box, there are three parameters that may be tuned to give improved accuracy for particular situations:**

**• m, the number of randomly selected predictor variables chosen at each node**

**• J, the number of trees in the forest**

**• tree size, as measured by the smallest node size for splitting or the maximum number of terminal nodes.”**

**We have considered these three hyper-parameters as follows:**

**• m: we control the number of variables as input by features selection, and we take the default value m=sqrt(number of features as input)**

**• J: this parameter is known not to cause over-fitting; see Breiman [31] (details are provided below)**

**• tree size: we control the maximal number of terminal nodes from 2 to 20 by steps of 2.**

**Notably, Breiman [31] states that “Using a random selection of features to split each node yields error rates that compare favorably to Adaboost (Y. Freund & R. Schapire, Machine Learning: Proceedings of the Thirteenth International conference, ∗∗∗, 148–156), but are more robust with respect to noise.”**

**2) *Over-fitting is not a problem with random forests, as far as the number of trees is concerned.***

**Random forests are allowed to have a considerable number of trees and this does not cause over-fitting. Indeed, from section 1.2 in Breiman [31]: “Section 2 gives some theoretical background for random forests. Use of the Strong Law of Large Numbers shows that they always converge so that overﬁtting is not a problem.” See in particular, Theorem 1.2 and the remark after.**

**3) *Equivalent models (in terms of their power to represent decision domains) might not be necessarily as easy to train.***

**From Breiman [31]: “Signiﬁcant improvements in classiﬁcation accuracy have resulted from growing an ensemble of trees and letting them vote for the most popular class.”**

**Indeed, a random forest can be viewed as a special case of the notion of “committee of experts”. It has been observed that it is easier *(i.e*., yielding better classification accuracy) to train (possibly loosely) several simple classifiers (*e.g*., randomly grown decision trees without pruning) and consider a vote over their individual decisions, than trying to train a single classifier (*e.g*., a single decision tree). See the following reference (sections 9.6 and 10.7) on this matter:**

**Bishop CM (1995) Neural networks for pattern recognition. Oxford University Press.**

**Other models could indeed be considered in principle, such as a multilayer perceptron (MLP), which can be viewed as a generalization of logistic regression models. It is known that MLP with only two hidden layers (but possibly with a large number of hidden nodes) is sufficiently powerful to represent arbitrarily complex decision domains (universality property). See section 4.2.2 of Bishop 1995. We think that random forests are as powerful as MLP for all practical purposes, provided decision domains are Lebesgue measurable sets, but with the advantage of being easier to train on datasets of small size.**

**As for deep learning models, development and evaluation of their performance in the context of the submitted work was not an objective for now. But we agree with the reviewer that future works could address this interesting topic, especially on a larger database.**

**To address the reviewer’s comment, we have added the following sentence at the end of Limitations (lines 619-620):**

**“Furthermore, other machine learning models than random forests could be assessed on a larger database.”**

2. As we can see from Table 2, the parameters chosen for each of the binary classification tasks vary. This begs the question of how practical this RF-based method is. Furthermore, it’s perceivable that with addition of new data in the future, the parameters will change, which means that the results presented here are far from conclusive.

**Response: We thank the reviewer for raising these important points.**

**1) Concerning the question “*This begs the question of how practical this RF-based method is*”, we do not see an objection. Indeed, several features are being considered and different combinations of them yield outputs to different dichotomous classification tasks. We do not expect different tasks to be handled with a single trained machine learning model, unless a multi-task model is being considered. The optimal combination of features used as input is part of a trained model. In any case, even if the same combination of features turned out to be optimal for various distinct tasks, one would still be using distinct trained algorithms for calculating the respective outputs. We do not see this fact as a lack of practical value, since outputs would be calculated with a computer program.**

**2) We agree with the statement that “*with addition of new data in the future, the parameters will change*”. We actually think that this point holds for any study exploring the potential of features in the presence of a relatively small sample size, including statistical models such as linear logistic regression and standard statistical tests. In our understanding, the issue here is the assumption of representativeness of datasets for the target population at stake, for both training and testing models (or hypotheses). In this context, it is indeed understood that increasing the sample size, whilst maintaining its representativeness, would yield more stable trained models and henceforth, stronger conclusions concerning the relevance of considered features and assessment of performance.**

**Nonetheless, as mentioned in our response to the reviewer’s previous comment, the main goal of the submitted study was to demonstrate the potential value of combining the proposed QUS features with pSWE feature. It is expected that refinement of the proposed model based on larger datasets might be needed for translation into clinical practice.**

**To address the reviewer’s comment, we have modified the following sentence in Limitations (lines 615-617):**

**“Further assessment would be required on a larger data set in a target population of patients with NAFLD or NASH, in particular to assess more precisely the impact of each of the proposed QUS features in the considered dichotomous tasks.”**

3. Minor comment: some of the text in the manuscript is in bold. Unclear if it follows any particular pattern. Recommend removing or cleaning up the bold formatting.

**Response: We had previously highlighted Figure and Table call outs in bold to facilitate the work by reviewers. We have removed the bold formatting to comply with the formatting requirements of PLOS One.**

6. PLOS authors have the option to publish the peer review history of their article ([what does this mean?](https://journals.plos.org/plosone/s/editorial-and-peer-review-process#loc-peer-review-history)). If published, this will include your full peer review and any attached files.


If you choose “no”, your identity will remain anonymous but your review may still be made public.


**Do you want your identity to be public for this peer review?** For information about this choice, including consent withdrawal, please see our [Privacy Policy](https://www.plos.org/privacy-policy).

Reviewer #1: No

Reviewer #2: No

Reviewer #3: No

[NOTE: If reviewer comments were submitted as an attachment file, they will be attached to this email and accessible via the submission site. Please log into your account, locate the manuscript record, and check for the action link "View Attachments". If this link does not appear, there are no attachment files.]

While revising your submission, please upload your figure files to the Preflight Analysis and Conversion Engine (PACE) digital diagnostic tool, <https://pacev2.apexcovantage.com/>. PACE helps ensure that figures meet PLOS requirements. To use PACE, you must first register as a user. Registration is free. Then, login and navigate to the UPLOAD tab, where you will find detailed instructions on how to use the tool. If you encounter any issues or have any questions when using PACE, please email PLOS at [figures@plos.org](mailto:figures@plos.org). Please note that Supporting Information files do not need this step.
