# Supplementary material for: Quantitative ultrasound, elastography, and machine learning for assessment of steatosis, inflammation, and fibrosis in chronic liver disease
Source: PLoS One. 2022 Jan 27;17(1):e0262291. doi: 10.1371/journal.pone.0262291 (PMC8794185; doi:10.1371/journal.pone.0262291)
Supplement: Attachment — Submitted filename: Response to Reviewers.docx [file pone.0262291.s008.docx]

PONE-D-21-21119R1

Quantitative ultrasound, elastography, and machine learning for assessment of steatosis, inflammation, and fibrosis in chronic liver disease

PLOS ONE

Dear Dr. Tang,

Thank you for submitting your manuscript to PLOS ONE. After careful consideration, we feel that it has merit but does not fully meet PLOS ONE’s publication criteria as it currently stands. Therefore, we invite you to submit a revised version of the manuscript that addresses the points raised during the review process.

Please submit your revised manuscript by Dec 10 2021 11:59PM. If you will need more time than this to complete your revisions, please reply to this message or contact the journal office at [plosone@plos.org](mailto:plosone@plos.org). When you're ready to submit your revision, log on to <https://www.editorialmanager.com/pone/> and select the 'Submissions Needing Revision' folder to locate your manuscript file.

Please include the following items when submitting your revised manuscript:

- A rebuttal letter that responds to each point raised by the academic editor and reviewer(s). You should upload this letter as a separate file labeled 'Response to Reviewers'.
- A marked-up copy of your manuscript that highlights changes made to the original version. You should upload this as a separate file labeled 'Revised Manuscript with Track Changes'.
- An unmarked version of your revised paper without tracked changes. You should upload this as a separate file labeled 'Manuscript'.

If you would like to make changes to your financial disclosure, please include your updated statement in your cover letter. Guidelines for resubmitting your figure files are available below the reviewer comments at the end of this letter.

If applicable, we recommend that you deposit your laboratory protocols in [protocols.io](http://protocols.io) to enhance the reproducibility of your results. Protocols.io assigns your protocol its own identifier (DOI) so that it can be cited independently in the future. For instructions see: <https://journals.plos.org/plosone/s/submission-guidelines#loc-laboratory-protocols>. Additionally, PLOS ONE offers an option for publishing peer-reviewed Lab Protocol articles, which describe protocols hosted on [protocols.io](http://protocols.io). Read more information on sharing protocols at <https://plos.org/protocols?utm_medium=editorial-email&utm_source=authorletters&utm_campaign=protocols>.

We look forward to receiving your revised manuscript.

Kind regards,

Omar Sultan Al-Kadi, PhD
Academic Editor
PLOS ONE

Journal Requirements:

Please review your reference list to ensure that it is complete and correct. If you have cited papers that have been retracted, please include the rationale for doing so in the manuscript text, or remove these references and replace them with relevant current references. Any changes to the reference list should be mentioned in the rebuttal letter that accompanies your revised manuscript. If you need to cite a retracted article, indicate the article’s retracted status in the References list and also include a citation and full reference for the retraction notice.

[Note: HTML markup is below. Please do not edit.]

Reviewers' comments:

Reviewer's Responses to Questions

**Comments to the Author**

1. If the authors have adequately addressed your comments raised in a previous round of review and you feel that this manuscript is now acceptable for publication, you may indicate that here to bypass the “Comments to the Author” section, enter your conflict of interest statement in the “Confidential to Editor” section, and submit your "Accept" recommendation.

Reviewer #1: All comments have been addressed

Reviewer #2: All comments have been addressed

Reviewer #3: All comments have been addressed

2. Is the manuscript technically sound, and do the data support the conclusions?

The manuscript must describe a technically sound piece of scientific research with data that supports the conclusions. Experiments must have been conducted rigorously, with appropriate controls, replication, and sample sizes. The conclusions must be drawn appropriately based on the data presented.

Reviewer #1: Yes

Reviewer #2: Yes

Reviewer #3: Yes

3. Has the statistical analysis been performed appropriately and rigorously?

Reviewer #1: Yes

Reviewer #2: Yes

Reviewer #3: Yes

4. Have the authors made all data underlying the findings in their manuscript fully available?

The [PLOS Data policy](http://www.plosone.org/static/policies.action#sharing) requires authors to make all data underlying the findings described in their manuscript fully available without restriction, with rare exception (please refer to the Data Availability Statement in the manuscript PDF file). The data should be provided as part of the manuscript or its supporting information, or deposited to a public repository. For example, in addition to summary statistics, the data points behind means, medians and variance measures should be available. If there are restrictions on publicly sharing data—e.g. participant privacy or use of data from a third party—those must be specified.

Reviewer #1: Yes

Reviewer #2: Yes

Reviewer #3: Yes

5. Is the manuscript presented in an intelligible fashion and written in standard English?

PLOS ONE does not copyedit accepted manuscripts, so the language in submitted articles must be clear, correct, and unambiguous. Any typographical or grammatical errors should be corrected at revision, so please note any specific errors here.

Reviewer #1: Yes

Reviewer #2: Yes

Reviewer #3: Yes

6. Review Comments to the Author

Please use the space provided to explain your answers to the questions above. You may also include additional comments for the author, including concerns about dual publication, research ethics, or publication ethics. (Please upload your review as an attachment if it exceeds 20,000 characters)

Reviewer #1: Thank you for addressing my comments. I do not have any further suggestions. Congratulations
As a minor note. The authors may consider adding this paper to their discussion as well (PMID: 32622685)

**Response to reviewer: As suggested, we have integrated reference PMID: 32622685 where appropriate in the Discussion:**

**«Recent works by Brattain et al, Durot et al., Kagadis et al., showed that fibrosis classification accuracy could be further improved, with AUCs in the range of 0.93 to 0.99, by using machine learning techniques such as automated image quality assessment and ROI selection [53], support vector machines [16] and deep learning techniques applied on shear wave elastography data [19].»**

Reviewer #2: (No Response)

Reviewer #3: This paper presented an ML-based method, specifically Random Forest (RF), to classify three important grades for the CLD population: steatosis, inflammation, and fibrosis. Feature sets of QUS and pSWE combind were compared against pSWE alone. When tested on a dataset of 82 patient, RF on combined feature sets outperformed pSWE alone.

The paper is clearly written and easy to follow. The dataset description is thorough, the experiment setup is clear, the results can be easily understood.

Major comments:
1. The selected feature set is different for each classification tasks. What are the overlapping features among these classification tasks?

**Response to reviewer: Thank you for the suggestion. Accordingly, we have added the following text:**

**“From Table 2, one observes that local ACS is the only common parameter in the best combinations of parameters for the three dichotomous classifications tasks of steatosis grade. Moreover, the three dichotomous classification tasks of the inflammation grade have no common parameters. For the four dichotomous classification tasks of the fibrosis stage, point shear wave elasticity is the only common parameter. However, except for the task F0 vs. F1-4, for which the best combination of parameters was pSWE,** $\boldsymbol{\mu}_{\boldsymbol{n}}$ **mean and** $\boldsymbol{1/(}\boldsymbol{\kappa+1)}$ **mean, there were three common parameters: pSWE,** $\boldsymbol{\mu}_{\boldsymbol{n}}$ **IQR and** $\boldsymbol{1/(}\boldsymbol{\kappa+1)}$ **IQR.”**

2. RF can also rank the weights of the selected features. Which features carry more weights? This is helpful to understand, because it can help interpret the ML results further.

**Response to reviewer: Thank you for the suggestion. For each of the selected combination of parameters corresponding to each dichotomous classification task, we computed the parameters’ importance with the R package “randomForest” based on the mean decrease in accuracy (*i.e*., with the option “type=1”). Accordingly, we have added Table S4, which reports the obtained importance values, in Supplementary Materials. We have added the following text:**

**“The importance values, viewed as mean decrease in accuracy, of each of the parameters within the combinations of parameters appearing in Table 2 are reported in Table S4, based on the R package “randomForest”.”**

3. One of the biggest limitations of the paper, as mentioned in the discussion, is that only RF is presented. SVM has been reported by other papers, it would be helpful to see how the RF classifier compares against SVM given the feature sets used.

**Response to reviewer: Thank you for the suggestion. Although the support vector machine (SVM) model offers in principle the same computational power as random forests (RF), the former model presents more hyper-parameters to tune than the latter. Indeed, whereas the RF model requires basically tuning the maximum number of terminal nodes, the SVM model requires a choice of basis (for projection of data in higher dimensional space) and the cost *C* for defining the soft margin. Since we had used the R package “randomForest” for random forests and we had programmed bootstrapping in R, we resorted to the R package “e1071” for SVM. We chose the default radial basis function (RBF) with the default degree 3 and RBF parameter γ set to its default value equal to the reciprocal of the number of parameters in each tested combination of parameters. Since the cost has an impact on the classification, we adopted the strategy of testing several powers of 2. Namely, we tested *C* = 2^n^ for n = 0, …, 10. In cases where the resulting AUC-ROC was maximal at *C* = 2^10^, we tested further powers of 2 until decrease in AUC.**

**Notice that if we had more available data, tuning the SVM with the help of a validation set would have allowed performing finer tuning, possibly based on a grid search over a range of hyper-parameters, which might allow for improvement on AUCs. However, considering the relatively small sample size (82 patients), we decided to stick to the 0.632+ bootstrap approach. Thus, we content in reporting the obtained results with the adopted strategy, based on 0.632+ bootstrap estimates of AUCs. Notice that we tested the combinations of parameters appearing in Table 2 for sake of comparison.**

**Accordingly, the resulting AUCs are reported in Table S5 in Supplemental Materials. We have added the following text:**

**“For sake of comparison, we present in Table S5 in Supplemental Materials the ROC-ACUs obtained with the support vector machine (SVM) model on the same combinations of parameters that are appearing in Table 2. For this purpose, we used the R package “e1071” (version 1.7-9, 2021) [43]. The kernel for projection of data in higher dimensional space was the radial basis function (RBF) of degree 3 with parameter “γ" equal to the reciprocal of the number of parameters (default values in the package). The ROC curves were produced with class weights of the form (p/N(0), (1-p)/N(1)), where N(0) and N(1) represent the number of elements in the two groups of a given dichotomous classification task, letting p vary from 0 to 1. As the cost *C* related to the soft margin of SVM has an impact on accuracy, powers of 2 (with exponent varying from 0 to 10) were tested with the 0.632+ bootstrap method, and the best obtained values are reported in Table S5. In cases where the best AUC was obtained with *C* = 2^10^, we tested further powers of 2 until decrease in ACU. Finer tuning may have been desirable for some classification tasks, notably for inflammation grade, but we did not pursue this task. As the number of hyper-parameters to be tuned for SVM is larger than for RF, further tuning of the SVM model might require a grid search on a validation set in the case of a larger sample size.”**

**Reference:**

**43: Meyer D, Dimitriadou E, Hornik K, Weingessel A, Leisch F, Chang CC, et al. Package ‘e1071’. Version 1.7-9. September 16, 2021.**

Minor comments:
1. The font size is different at a number of locations, e.g., Line 279, 281, 302, 343, 526, etc.

**Response to reviewer: We have harmonized font size to 12 points throughout the manuscript.**

2. Figures appear blurry, but that could be the result of how the pdf is generated.

**Response to reviewer: The figures appear blurry in the rendered PDF but they are fine in the submitted TIFF and PDF files**

7. PLOS authors have the option to publish the peer review history of their article ([what does this mean?](https://journals.plos.org/plosone/s/editorial-and-peer-review-process#loc-peer-review-history)). If published, this will include your full peer review and any attached files.

**Response to reviewer: You may include the peer review history of this article.**

If you choose “no”, your identity will remain anonymous but your review may still be made public.


**Do you want your identity to be public for this peer review?** For information about this choice, including consent withdrawal, please see our [Privacy Policy](https://www.plos.org/privacy-policy).

Reviewer #1: No

Reviewer #2: No

Reviewer #3: No

[NOTE: If reviewer comments were submitted as an attachment file, they will be attached to this email and accessible via the submission site. Please log into your account, locate the manuscript record, and check for the action link "View Attachments". If this link does not appear, there are no attachment files.]

While revising your submission, please upload your figure files to the Preflight Analysis and Conversion Engine (PACE) digital diagnostic tool, <https://pacev2.apexcovantage.com/>. PACE helps ensure that figures meet PLOS requirements. To use PACE, you must first register as a user. Registration is free. Then, login and navigate to the UPLOAD tab, where you will find detailed instructions on how to use the tool. If you encounter any issues or have any questions when using PACE, please email PLOS at [figures@plos.org](mailto:figures@plos.org). Please note that Supporting Information files do not need this step.
